# Longitudinally and Vertically Balanced Sparse Indoor Temperature–Humidity Sensing Preserves Threshold-Defined Risk Inference in a Chinese Solar Greenhouse

**DOI:** 10.3390/s26206391

**Published:** 2026-10-09

**Authors:** Junjie Shi, Jinping Li, Yanhong Wei, Chong Zhang, Ya Ma, Wenjie Yang, Zhiwei Fan

**Affiliations:** 1 School of Energy and Power Engineering, Lanzhou University of Technology, Lanzhou 730050, China; 2 Key Laboratory of Multi-Supply System with Solar Energy and Biomass, Lanzhou University of Technology, Lanzhou 730050, China; 3 China Northwest Collaborative Innovation Center of Low-Carbon Urbanization Technologies, Lanzhou 730050, China; 4 School of Energy and Power Engineering, Lanzhou Petrochemical University of Vocational Technology, Lanzhou 730060, China; 5 School of Green Energy and Storage, Lanzhou University of Technology, Lanzhou 730050, China; 6 School of Mechanical Engineering, Lanzhou Petrochemical University of Vocational Technology, Lanzhou 730060, China; 7 Technical Institute of Physics and Chemistry, Chinese Academy of Sciences, Beijing 100190, China; 8 School of Petroleum and Chemical Engineering, Lanzhou Petrochemical University of Vocational Technology, Lanzhou 730060, China; 9 Department of Infrastructure Development and Support, Tian Hua Institute of Chemical Machinery & Automation Co., Ltd., Lanzhou 730060, China

**Keywords:** greenhouse monitoring, smart agriculture, sensor networks, sparse sensing, longitudinal and vertical coverage, temperature and relative humidity, agronomic risk, whole-sensor holdout, selective inference

## Abstract

Sensor failures reduce the spatial information available for greenhouse microclimate monitoring, but their practical consequences depend on whether agronomic risk decisions remain reliable. We evaluated the progressive loss of paired indoor temperature–relative humidity (T/RH) sensors in a Chinese solar greenhouse using whole-sensor holdout. Longitudinally and vertically balanced subsets and transparent estimators were frozen before a sealed final test of 28 dates, four calendar blocks and six stage-aware risks. Relative to a 20-input reference, a longitudinally and vertically balanced six-input network increased the temperature mean absolute error by 0.014 °C and the relative-humidity error by 0.027 percentage points; macro-recall changed by −0.001 and support-aware macro-F1 by −0.004, while median false-negative exposure remained unchanged. The results remained within the predefined limits across 26 agricultural-threshold variants. In an exploratory validation-only comparison, spatially concentrated six-input layouts had greater temperature error (+0.080 °C, 95% block-bootstrap interval +0.058 to +0.104 °C), relative-humidity error (+0.099 percentage points), and macro-F1 loss (−0.004) than longitudinally and vertically balanced layouts. Macro-F1 did not differ consistently between the longitudinally and vertically balanced layouts and the unconstrained random layouts. Longitudinal and vertical coverage therefore functions as a robustness constraint against concentrated input loss in this system, rather than evidence of universal six-sensor sufficiency or cross-greenhouse generalisation.

## 1. Introduction

Smart greenhouse operation depends on measurements that represent conditions around the crop and remain useful when some sensors are unavailable. Air temperature, relative humidity, and vapour pressure deficit (VPD) vary across greenhouse length, height, and time. Distributed sensing and spatial mapping studies have documented this variation, including local conditions related to condensation and crop stress [1,2,3,4]. Recent multi-year measurements in a commercial tomato greenhouse showed persistent horizontal temperature and VPD gradients, while a study of Chinese solar greenhouses likewise reported spatial, vertical, and temporal non-uniformity [5,6]. Additional field and modelling studies have shown that greenhouse microclimates can remain non-uniform across length and height as solar load, crop transpiration, crop height, ventilation, and greenhouse structure change [7,8,9,10]. Fine-scale temperature monitoring further shows that sensor position can materially affect measured temperature and temperature-derived crop indices [11]. Denser networks can describe this variation more fully, but they also require more installation, maintenance, and fault management.

Complete sensor failure removes information from one location rather than merely adding noise. A missing temperature–relative humidity (T/RH) pair affects temperature and humidity reconstruction and the risk decisions derived from them. These decisions include low temperature, high humidity, vapour-pressure-deficit (VPD) stress, and near-condensation potential. Their agricultural meaning also depends on crop stage and light conditions, and excessive VPD can impair plant water status and productivity [12,13,14,15]. Average reconstruction error alone therefore cannot show whether a degraded network preserves an agronomically relevant decision. A small numerical error may cross a decision threshold, whereas a larger error may leave the decision unchanged.

Recent greenhouse research has pursued three related routes. Sensor-placement studies select compact subsets by minimising continuous error, and fixed-layout models forecast representative temperature and humidity states [16,17,18,19]. Greenhouse fault-tolerant and missing-data studies reconstruct faulty or absent environmental readings and report continuous prediction error [12,20,21]. Related environmental-sensor research has also learned spatiotemporal relations among network nodes for missing-value recovery [22]. A recent digital-twin framework dynamically selected informative sensor locations and validated the subsets using the Z-index, mean absolute error (MAE), and root mean squared error (RMSE) [23]. These advances improve monitoring efficiency or continuity, but their principal endpoints are continuous reconstruction, forecasting, or control-oriented aggregate states. They do not directly test whether the remaining sensors preserve threshold-defined agronomic risk decisions after a complete paired sensor is lost. Three questions therefore matter: which sensor is missing, how many non-target inputs remain, and whether those inputs still cover the greenhouse length and height. A leakage-safe evaluation must withhold the complete target sensor, fix the functioning-input count, and apply the same spatial rule to every degraded network.

Here, we progressively reduced an indoor T/RH network in a Chinese solar greenhouse. For each held-out target pair, K is the number of functioning non-target input pairs. We evaluated K = 20, 16, 12, 8, and 6 using target-specific subsets that retained coverage of four longitudinal sections and three sensor heights. The primary question was whether six stage-aware risk decisions remained close to a 20-input reference, not whether continuous reconstruction error remained small. The six decisions were low temperature, high temperature, high RH, near-condensation potential, low VPD, and high VPD. Robustness was assessed using 26 predefined threshold variants, calendar-block stability, risk-specific safeguards, and a development-calibrated 90th-percentile (q90) abstention rule. The sealed final test contained 28 dates in four calendar blocks and was opened only after the estimation policy and acceptance criteria were frozen.

The new approach is therefore evaluative rather than a new prediction architecture: it treats agronomic threshold decisions as the primary endpoints and combines complete target-sensor holdout, fixed input-count comparisons, longitudinal and vertical coverage constraints, stage-aware risk definitions, predefined threshold sensitivity, calendar-block checks, and support-aware selective inference. This bounded framework tests whether a longitudinally and vertically balanced six-input network can preserve threshold-defined risk decisions after loss of a complete paired sensor and reports when that conclusion becomes unreliable. The result is a tested condition for this greenhouse and failure scenario. It is not evidence that any six-sensor layout is sufficient, and the study does not evaluate yield, disease, energy use, or closed-loop control.

## 2. Materials and Methods

### 2.1. Study Setting and Indoor Sensing Network

The study used an indoor temperature–relative humidity network in a northern Chinese solar greenhouse located in Mengjiashan Village, Xigu District, Lanzhou, Gansu Province, China (103.647° E, 36.064° N; altitude 1780.7 m). The greenhouse had a three-wall, single-slope configuration, an approximate length of 50 m, span of 6.0 m, ridge height of 3.2 m, and eave height of 2.7 m. Air temperature and relative humidity were measured using VMS-3002-WS-V10 temperature–humidity transmitters (VEMSEE, Jinan, China). All transmitters were purchased from the same manufacturer as one procurement batch and installed as one network. No independent pre-installation co-location calibration was performed; therefore, measurement comparability was based on the common model, procurement batch, and manufacturer’s specifications. The supplied specification reports an accuracy of ±0.5 °C at 25 °C for temperature and ±3%RH at 60%RH and 25 °C for RH; the probe operating range was −40 to 80 °C and 0–100%RH. The retained air-sensor catalogue for the SCI-2 holdout analysis contained 22 indoor T/RH pairs distributed across the 10, 20, 30, and 40 m sections and three nominal heights (0.7, 1.2, and 1.7 m). Wall-surface pairs at 0 and 50 m were excluded from the ordinary crop-air states. Each retained target pair was evaluated as a possible complete whole-sensor failure.

Before application of the fixed health-cycle coverage criterion, the indoor monitoring layout comprised 24 paired T/RH probes arranged at four longitudinal sections (10, 20, 30, and 40 m), with six transverse and vertical positions (A–F) in each section. All probes were enclosed in louvred radiation shields that reduced direct solar-radiation exposure while permitting air exchange. The nominal measurement heights were 0.7, 1.2, and 1.7 m. Tomato canopy height varied approximately from 1.3 to 1.5 m during the analysed period, placing the highest sensor level approximately 0.2–0.4 m above the canopy top. The upper ventilation opening was approximately 3.2 m above ground, or about 1.5 m above the highest sensor level. These dimensions describe the mounting context; airflow effects were not independently quantified. Of the 24 installed pairs, 20A had no valid paired observations and 20C retained 22.9% of the expected five-minute observations, below the predefined 75% cycle-coverage criterion. Both were excluded before the holdout analysis, leaving the fixed 22-pair reference catalogue. Full coordinates and sensor metadata are provided in Supplementary Table S4.

### 2.2. Data Roles and Local Processing

The analysis used indoor paired T/RH observations, sensor-position metadata, date–role assignments, the tomato stage schedule, the target-specific subset catalogue, and development-calibrated q90 residual margins. Measurements were represented on a local Asia/Shanghai 5 min time grid. The source stage schedule defined fruit set from 11 February to 31 March and fruiting from 1 April to 30 June; the final test used the prespecified date–role assignments within these stage definitions. The final test contained 28 dates labelled as test before observation loading. Outdoor temperature, outdoor RH, radiation, and control-state variables were excluded from the final-test computation. Existing project processing was reused rather than reprocessing raw exports: cumulative exports were deterministically deduplicated, co-located T/RH channels were paired, temporal gaps were retained without interpolation, known non-crop-air channels were excluded, and the SCI-2-specific variables and risk flags were then recalculated on the 5 min grid. All processing was performed locally on the project workstation.

### 2.3. Whole-Sensor Holdout and Progressive Network Design

For each trial, one retained target sensor pair was designated as completely unavailable before estimation. Whole-sensor holdout is therefore an evaluation condition, not a fault-detection algorithm. All observations from the designated target were excluded from estimator inputs, and the analysis did not estimate the failure onset or cause. It assumes that an upstream monitoring or maintenance process has already identified the unavailable pair. The pre-analysis exclusions of 20A and 20C were coverage-eligibility decisions, not an online failure-detection rule. K denotes the exact number of functioning non-target input pairs; the evaluated levels were K = 20, 16, 12, 8, and 6.

**Figure 1:**
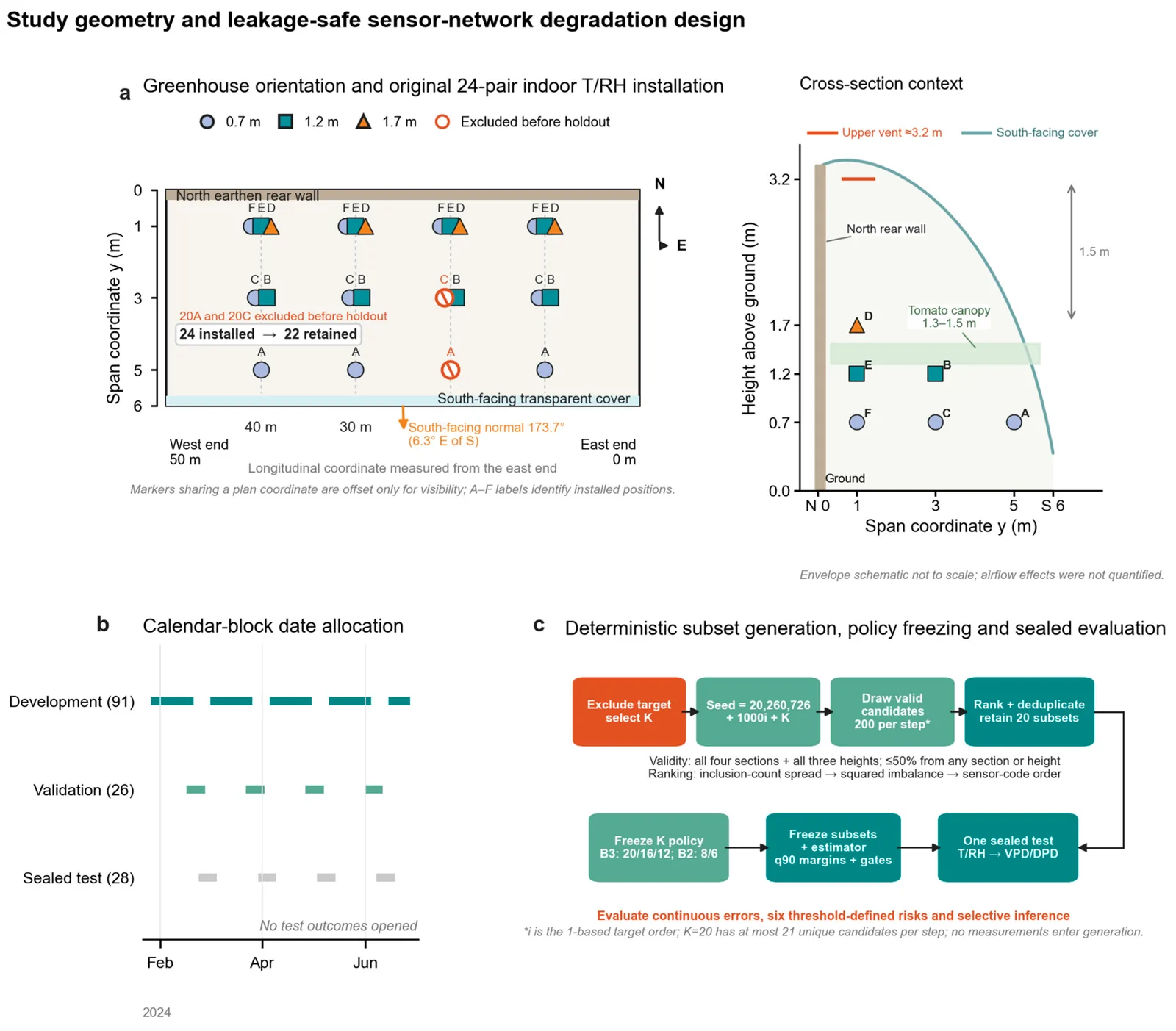
Orientation-aware study geometry and leakage-safe sensor-network degradation design. (**a**) North-up plan and schematic cross-section of the Chinese solar greenhouse. The plan shows the north rear wall, south-facing transparent cover, east end (0 m), west end (50 m), measured south-facing normal (azimuth 173.7°, 6.3° east of south), and all 24 originally installed paired temperature–relative humidity (T/RH) probes. Marker shapes and colours identify nominal heights of 0.7, 1.2, and 1.7 m, as indicated in the panel legend. Probes 20A and 20C, which failed the prespecified pre-holdout coverage screen, are shown as open slashed symbols, leaving the fixed 22-pair reference network. The cross-section shows positions A–F, the reported tomato canopy height (approximately 1.3–1.5 m), and the upper-vent level (approximately 3.2 m). The envelope is schematic and not to scale; airflow effects were not independently quantified. (**b**) Calendar-block allocation of eligible dates to development (teal), validation (sage), and sealed-test (grey) sets. (**c**) Reproducible leakage-safe workflow. After target exclusion and selection of K, target–K-specific seeds generate valid longitudinally and vertically constrained candidates, which are ranked and deduplicated to retain 20 subsets. The K-specific estimator policy, target-specific subsets, q90 margins and acceptance gates are frozen before the sealed test is opened once. Temperature and RH are then reconstructed, VPD and DPD are derived, and continuous errors, six threshold-defined risks and selective inference are evaluated. Arrows indicate workflow progression. Orange highlights target exclusion and evaluation; sage and teal distinguish the preparation and frozen-evaluation steps. No target observations or measurement values enter subset generation. K denotes the number of functioning non-target input pairs; q90 denotes the development-calibrated 90th-percentile residual margin; VPD, vapour pressure deficit; DPD, dew-point depression.

The 20 subsets for each target–K combination were generated deterministically from sensor metadata only; no measurement values were read or scored. Targets followed the fixed 1-based order i = 1, …, 22 in the holdout catalogue, and the random-number-generator seed was 20,260,726 + 1000i + K. At each of the 20 sequential selection steps, the algorithm attempted to collect 200 unique valid candidates not selected previously, with a maximum of 4000 random draws per step (for K = 20, only 21 distinct size-20 subsets can exist among the 21 non-target pairs). A candidate was valid only if it included all four longitudinal sections (10, 20, 30, and 40 m), all three nominal heights (0.7, 1.2, and 1.7 m), and no section or height supplied more than 50% of its inputs. No transverse-position coverage constraint was imposed. Valid candidates were ranked first by the prospective spread between the largest and smallest cumulative input-inclusion counts, second by the sum of squared deviations from the ideal cumulative inclusion count sK/21 at selection step s, and third by the concatenated sensor-code order as a deterministic tie-breaker. The top-ranked candidate was retained, cumulative counts were updated, and the process was repeated until 20 unique subsets were fixed. Across all 22 targets and five K levels, this produced 2200 target-specific subsets; the maximum between-input inclusion-count spread within a target–K group was two. The catalogue was frozen before final-test opening (Table 1 and Figure 1).

### 2.4. Exploratory Validation-Only Comparison of K = 6 Layout Geometry

After the original final-test protocol was frozen, we performed a separate exploratory comparison to test whether the longitudinal and vertical coverage constraint was associated with better K = 6 performance. This comparison used only validation dates and did not access final-test observations. For each of the 22 held-out target pairs, we compared 20 prespecified longitudinally and vertically balanced K = 6 subsets with 20 deterministic unconstrained random K = 6 subsets and 20 deterministic spatially concentrated K = 6 subsets. The spatially concentrated subsets spanned no more than two greenhouse sections or a single nominal height. Concentration was therefore defined by longitudinal section or nominal height; transverse position was not used as a balancing criterion in any layout family. All layouts excluded the held-out target pair and used the same frozen K = 6 whole-network-median estimator.

For each target, date, and layout family, we first summarised the 20 layout replicates by their median performance. We then calculated the paired differences between each comparator family and the longitudinally and vertically balanced family. Temperature and RH mean absolute error, six-risk macro-recall, and six-risk macro-F1 were summarised at the level of the four prespecified seven-day validation blocks. Uncertainty was estimated by resampling calendar blocks with replacement (20,000 percentile bootstrap replicates); individual 5 min observations were not resampled. Positive effects in the layout-comparison figure denote degradation of the comparator relative to the longitudinally and vertically balanced layout. This analysis was used to assess the interpretation of the longitudinal and vertical coverage constraint and is not part of the prespecified final-test confirmation.

### 2.5. Transparent Estimation Policy

Candidate transparent estimators were compared within the development/validation workflow, and the estimator for each K level was selected before final-test access. The resulting K-dependent policy used the current same-height median (B3) at K = 20, 16, and 12 and the current whole-network median (B2) at K = 8 and 6. This complete mapping, together with the target-specific subsets, development-calibrated q90 margins and prespecified acceptance gates, was then frozen. No test-time method re-ranking, refitting, new subset generation, or complex-model replacement was allowed. The final test therefore evaluated the prespecified network-size policy rather than the best method discovered after test opening.

### 2.6. Derived Microclimate Variables and Risk Definitions

Temperature and RH were reconstructed directly. VPD and dew-point depression (DPD) were derived from reconstructed and true T/RH values using the frozen project formulas [24,25]. Saturation vapour pressure was calculated as e_s(T) = 0.6108 exp [17.27T/(T + 237.3)] kPa, and VPD = e_s(T)(1 − RH/100). Dew-point temperature was calculated using γ = ln(RH/100) + 17.27T/(237.3 + T) and T_dew = 237.3γ/(17.27 − γ). DPD was then calculated as DPD = T − T_dew. Here, T is air temperature (°C), RH is relative humidity (%), e_s is saturation vapour pressure (kPa), T_dew is dew-point temperature (°C), and γ is dimensionless. RH was numerically bounded to 0.1–100% only for the dew-point calculation, reconstructed RH was bounded to 0–100%, and VPD was constrained to non-negative values. The six evaluated risks were low temperature, high temperature, high RH, near-condensation potential, low VPD, and high VPD. Stage-specific primary temperature ranges were 17–26 °C for fruit set and 18–27 °C for fruiting; these operating ranges were prespecified project decision rules informed by two tomato-production textbooks [26,27] (Supplementary Table S1). Stage-specific VPD operating bands were 0.8–1.2 kPa and 0.8–1.3 kPa, respectively. They were prespecified operational risk-screening bands, not assertions of a universal all-day optimum: published tomato response functions vary with developmental stage and sun, cloud, and night conditions [13,14]. The fruiting upper band of 1.3 kPa therefore functions as a high-VPD screening boundary whose applicability is evaluated by the predefined sensitivity analysis, rather than as an invariant physiological optimum. RH >85% was treated as a high-humidity risk screen, rather than a diagnosis of disease; tomato studies have associated sustained RH above this level with conditions conducive to grey-mould infection and have identified prolonged high RH as critical for fruit set under high temperature [28,29]. DPD ≤ 2 °C was treated as a conservative near-condensation-potential screen, informed by an empirical greenhouse leaf-wetness model that used a 2 °C dew-point threshold [30]. These thresholds were not re-estimated from the final test.

### 2.7. Metrics

Continuous reconstruction was assessed using median daily target-subset MAE for temperature and RH; VPD and DPD MAE were secondary derived-variable diagnostics. Forced risk decisions were assessed using six-risk macro-recall, support-aware macro-F1, and median false-negative risk-exposure duration, together with risk-specific recall, support-aware F1, and false-negative exposure. Macro values were unweighted across the six risks. Selective inference was assessed using q90 retained-decision coverage, retained-decision performance, and rejected true-risk exposure. For each K, candidate estimator, and directly reconstructed variable, q90 margins were calibrated only from development observations by pooling a deterministic sample of up to 512 evenly spaced valid absolute residuals from each target-subset evaluation and taking the 90th percentile. At evaluation, symmetric temperature and RH intervals were formed around each estimate, with RH limits clipped to 0–100%; VPD and DPD bounds were obtained by propagating the four temperature–RH interval corners through the frozen formulas. A risk decision was retained only when the full propagated interval supported the same positive or negative label; otherwise, the decision was rejected. Coverage was the number of retained decisions divided by the number of truth-valid bins. To make class support explicit, Supplementary Table S2 reports the valid and true-positive 5 min bins for every primary risk rule and crop stage, after removing duplicate subset evaluations. For each risk, support-aware F1 was calculated as 2TP/(2TP + FP + FN), where TP, FP and FN denote true positives, false positives and false negatives, respectively. A target-date-risk unit with true positives but no predicted positives therefore received F1 = 0 when the denominator was positive; units with no positive support in either truth or prediction did not contribute to that risk-specific F1 aggregation [31].

### 2.8. Final-Test Design and Prespecified Gates

The final test comprised four non-overlapping, temporally separated seven-day blocks: B05 (fruit set, 26 February 2024 to 3 March 2024), B10 (fruiting, 1 April 2024 to 7 April 2024), B15 (fruiting, 6 May 2024 to 12 May 2024) and B20 (fruiting, 10 June 2024 to 16 June 2024). This block structure retained calendar-separated evaluation units across the observed period and enabled block-level stability checks instead of treating all 28 dates as one undifferentiated test period. The blocks were not treated as independent experimental replicates. K = 6 was confirmed only if its absolute usability gates, relative K = 6 versus K = 20 preservation gates, risk-specific safeguards, threshold-robustness checks and block-stability rules passed. Selective inference was judged separately. These specifications were defined in a dated internal final-test protocol before final-test observations were loaded; the protocol and execution manifest can be made available to editors and reviewers under controlled access.

The acceptance limits were frozen before the sealed test as study-specific engineering decision criteria rather than as universal crop-damage thresholds. The absolute temperature and RH error limits were informed by the nominal accuracy of the installed sensors at the manufacturer’s stated reference conditions and by the scale of errors reported in greenhouse monitoring and reconstruction studies [32]. This comparison provides scale context and is not evidence of independent calibration. The recall, support-aware F1, missed-exposure and relative-preservation limits were author-defined guardrails intended to prevent small average reconstruction errors or macro-averages from concealing loss of threshold-defined risk information. Their numerical values are therefore reported as prespecified tolerances, not as externally standardised agricultural cut-offs. The basis and outcome of each gate family are reported in Supplementary Table S3. Dependence on the agricultural label definitions was evaluated separately across 26 prespecified threshold variants (Supplementary Table S5).

### 2.9. Statistical Uncertainty and Sensitivity Analyses

To characterise uncertainty in the final-test K = 6 versus K = 20 comparison without pseudo-replication, we calculated one paired K = 6-minus-K = 20 difference for each of the 28 eligible test dates. For each K and date, temperature and RH MAE were medians across the applicable target-subset evaluations. Recall and support-aware F1 were first summarised as medians within each risk and then averaged with equal weight across the six risks; false-negative exposure was the median of the six risk-specific summaries. We estimated 95% percentile intervals using 20,000 deterministic bootstrap resamples (seed 20,260,905): a stratified-date bootstrap resampled dates within each of the four calendar blocks, and a whole-block cluster bootstrap resampled the four complete seven-day blocks. Two-sided exact sign tests used the non-zero paired daily differences. Leave-one-block-out sensitivity recalculated the median paired difference after omitting each complete block. The date was the inferential unit; 5 min bins, target pairs and subset replicates were not treated as independent samples. These difference-from-zero analyses were sensitivity checks, not tests of equivalence or non-inferiority, and they did not replace the prespecified acceptance gates.

### 2.10. Reproducibility and Audit Trail

The final run produced machine-readable curves for all frozen methods, risk-specific tables, q90 summaries, threshold-sensitivity tables, block-stability tables, gate results, and a hash-based execution manifest. The main daily table contained 246,400 rows, and the threshold-sensitivity table contained 960,960 rows. Analysis scripts were run in Python 3.14.5 on macOS 26.5.2. Aggregated source tables underlying the main figures and Supplementary Table S2 were retained with the project materials; the raw observations and access-dependent code are available to editors and reviewers under controlled access. The formal result was computed locally after a successful metadata-only dry run; a reporting-only dependency issue was corrected without rereading observations or recalculating scientific outputs. OpenAI Codex (GPT-5) was used for language editing, code organisation, and figure-layout refinement; no raw project observations were uploaded to an external service, and all outputs were checked and approved by the authors.

## 3. Results

### 3.1. A Longitudinally and Vertically Balanced Degradation Experiment Was Evaluated Without Target Leakage

We evaluated progressive loss of indoor temperature–relative humidity (T/RH) sensing under a whole-sensor holdout design. For each held-out target sensor pair, the functioning-input count, K, represented the exact number of non-target sensor pairs available to the estimator. The tested levels were K = 20, 16, 12, 8, and 6, with the transparent estimator selected before opening the sealed test set: the same-height median was used at K = 20, 16, and 12, whereas the whole-network median was used at K = 8 and 6. The final test comprised 28 dates distributed across four non-overlapping, temporally separated seven-day calendar blocks, including one fruit-set block and three fruiting blocks. The final daily evaluation table contained 246,400 method-level records, and the threshold-sensitivity analysis contained 960,960 records. All primary test checks, including test-role isolation, finite metrics, curve completeness, and database integrity, passed.

### 3.2. Longitudinal and Vertical Coverage Reduced Degradation Caused by Concentrated K = 6 Layouts in Validation

**Figure 2:**
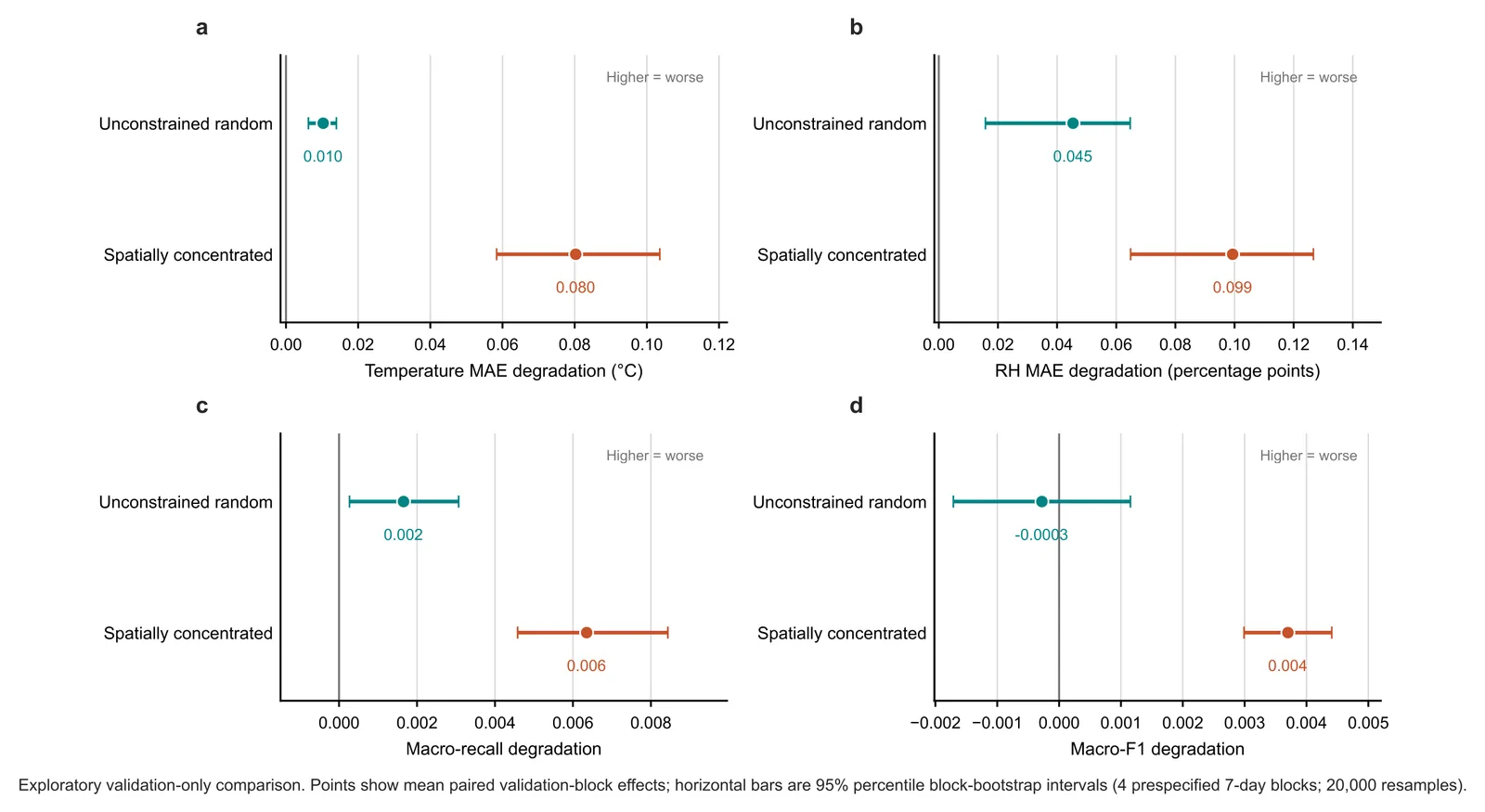
Longitudinal and vertical coverage reduces degradation under concentrated K = 6 layouts. Exploratory validation-only comparisons of longitudinally and vertically balanced K = 6 layouts with unconstrained random and spatially concentrated K = 6 layouts for (**a**) temperature mean absolute error (MAE), (**b**) relative humidity (RH) MAE, (**c**) six-risk macro-recall, and (**d**) six-risk macro-F1. Teal and orange points and bars indicate unconstrained random and spatially concentrated layouts, respectively. Points show mean paired effects across four prespecified seven-day validation blocks after taking the median across 20 layouts for each target-date-layout family. Horizontal bars show 95% percentile intervals from 20,000 block-bootstrap resamples. Positive values indicate degradation of the comparator relative to the longitudinally and vertically balanced layout. The comparison did not access final-test observations.

The exploratory validation-only comparison distinguished longitudinal and vertical coverage from the sensor count. Relative to the longitudinally and vertically balanced K = 6 layouts, spatially concentrated K = 6 layouts had higher mean temperature MAE by 0.080 °C (95% block-bootstrap interval, 0.058–0.104 °C) and higher RH MAE by 0.099 percentage points (0.065–0.127). They also had lower macro-recall by 0.006 (0.005–0.008) and lower macro-F1 by 0.004 (0.003–0.004). The direction of degradation was consistent across the four validation blocks (Figure 2).

Unconstrained random K = 6 layouts showed smaller increases in reconstruction error than spatially concentrated layouts, but their errors remained higher than those of longitudinally and vertically balanced layouts: temperature MAE increased by 0.010 °C (0.006–0.014) and RH MAE by 0.045 percentage points (0.016–0.065). Macro-recall was also modestly lower (0.002; 0.0003–0.003). However, the macro-F1 comparison was inconclusive because the comparator-degradation interval crossed zero (−0.0003; −0.002 to +0.001). Thus, the evidence does not support a claim that longitudinally and vertically balanced layouts uniformly outperform ordinary random layouts in every threshold-defined metric. Rather, it shows that the longitudinal and vertical coverage constraint protects against the more substantial degradation introduced by concentrated layouts.

### 3.3. Six Functioning Inputs Preserved Continuous Reconstruction and Forced Risk Decisions

**Figure 3:**
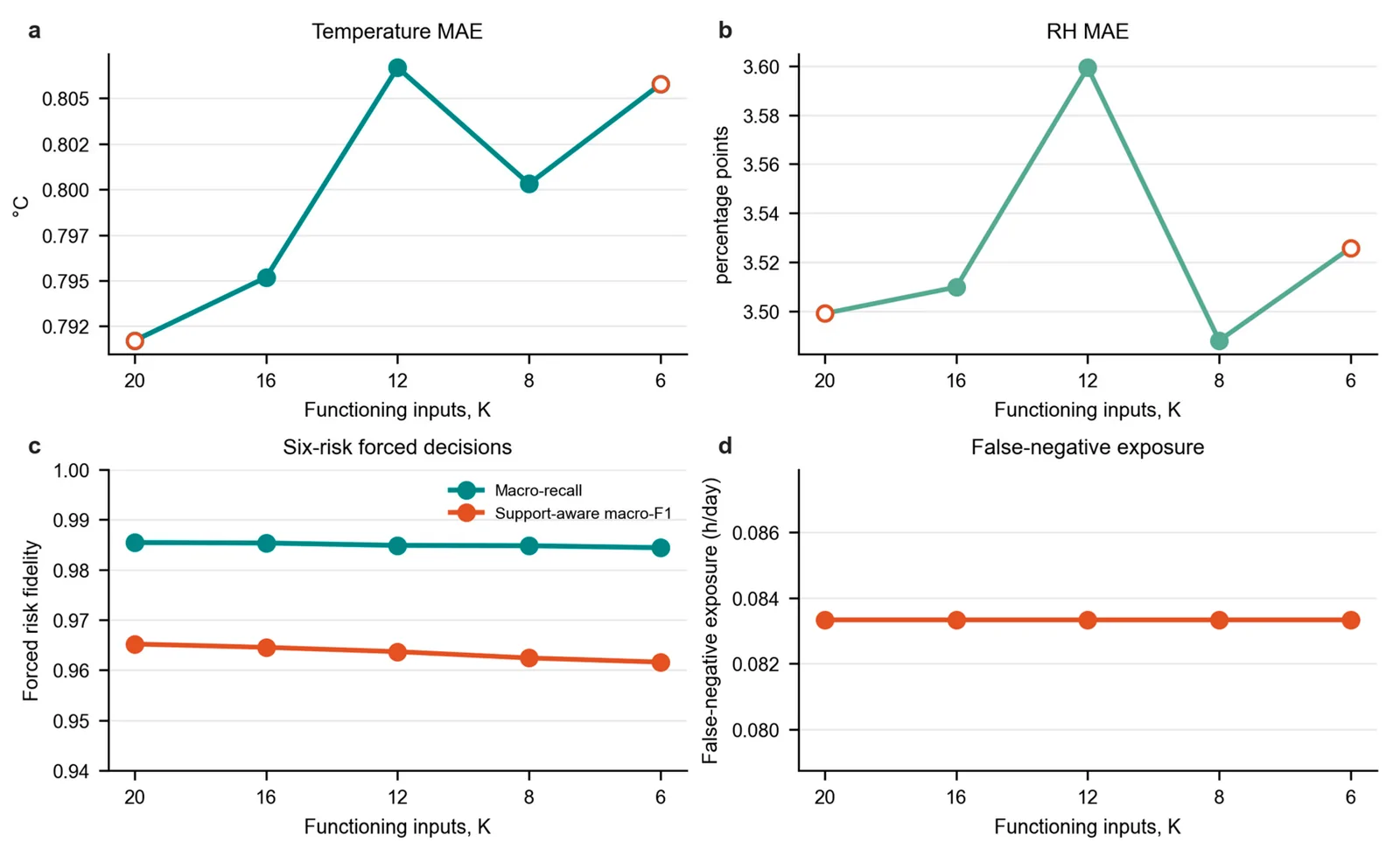
Final-test reconstruction and risk-fidelity curves across frozen functioning-input counts K. (**a**) Median temperature mean absolute error (MAE; teal). (**b**) Median relative humidity (RH) MAE (sage). Open orange-outlined markers in (**a**,**b**) highlight K = 20 and K = 6. (**c**) Six-risk forced macro-recall (teal) and support-aware macro-F1 (orange). (**d**) Median false-negative risk-exposure duration (orange).

The final test confirmed that the selected K = 6 network remained close to the K = 20 reference network for both continuous reconstruction and agronomic risk classification. The temperature MAE increased from 0.792 °C at K = 20 to 0.806 °C at K = 6, an absolute increase of 0.014 °C. The RH MAE increased from 3.499 to 3.526 percentage points, an increase of 0.027 percentage points. The corresponding VPD and dew-point-depression MAE values were 0.156 and 0.953 at K = 20, compared with 0.175 and 0.983 at K = 6, respectively. The final-test reconstruction and risk-fidelity curves are shown in Figure 3, and the complete primary comparison is reported in Table 2.

The small continuous errors translated into similarly small changes in the forced risk decisions. Macro-recall changed from 0.985 at K = 20 to 0.984 at K = 6, whereas support-aware macro-F1 changed from 0.965 to 0.962. The K = 6 minus K = 20 differences were therefore −0.001 for macro-recall and −0.004 for support-aware macro-F1. The median false-negative exposure remained 0.083 h/day for both network sizes. These values satisfied all prespecified absolute usability gates and all relative preservation gates, confirming K = 6 as the primary final-test outcome rather than invoking the prespecified fallback to K = 8 or K = 12.

The paired date-level sensitivity analysis gave median K = 6-minus-K = 20 differences of +0.010 °C for temperature MAE, +0.012 percentage points for RH MAE, −0.001 for macro-recall, −0.003 for support-aware macro-F1, and 0.000 h/day for median false-negative exposure. The corresponding stratified-date 95% percentile intervals were −0.040 to +0.030 °C, −0.065 to +0.151 percentage points, −0.002 to +0.002, −0.005 to 0.000, and 0.000 to +0.042 h/day, respectively. Two-sided exact sign-test *p* values were 0.851, 0.851, 0.701, 0.122, and 0.332. Whole-block cluster-bootstrap intervals and leave-one-block-out ranges are reported in Supplementary Table S7. The leave-one-block-out support-aware macro-F1 difference ranged from −0.005 to −0.001. None of the exact sign tests yielded *p* < 0.05, but absence of a difference-from-zero result is not evidence of equivalence or non-inferiority; preservation remains defined by the frozen acceptance limits.

### 3.4. Risk-Specific Performance Remained High, but Conservative Coverage Varied by Risk

**Figure 4:**
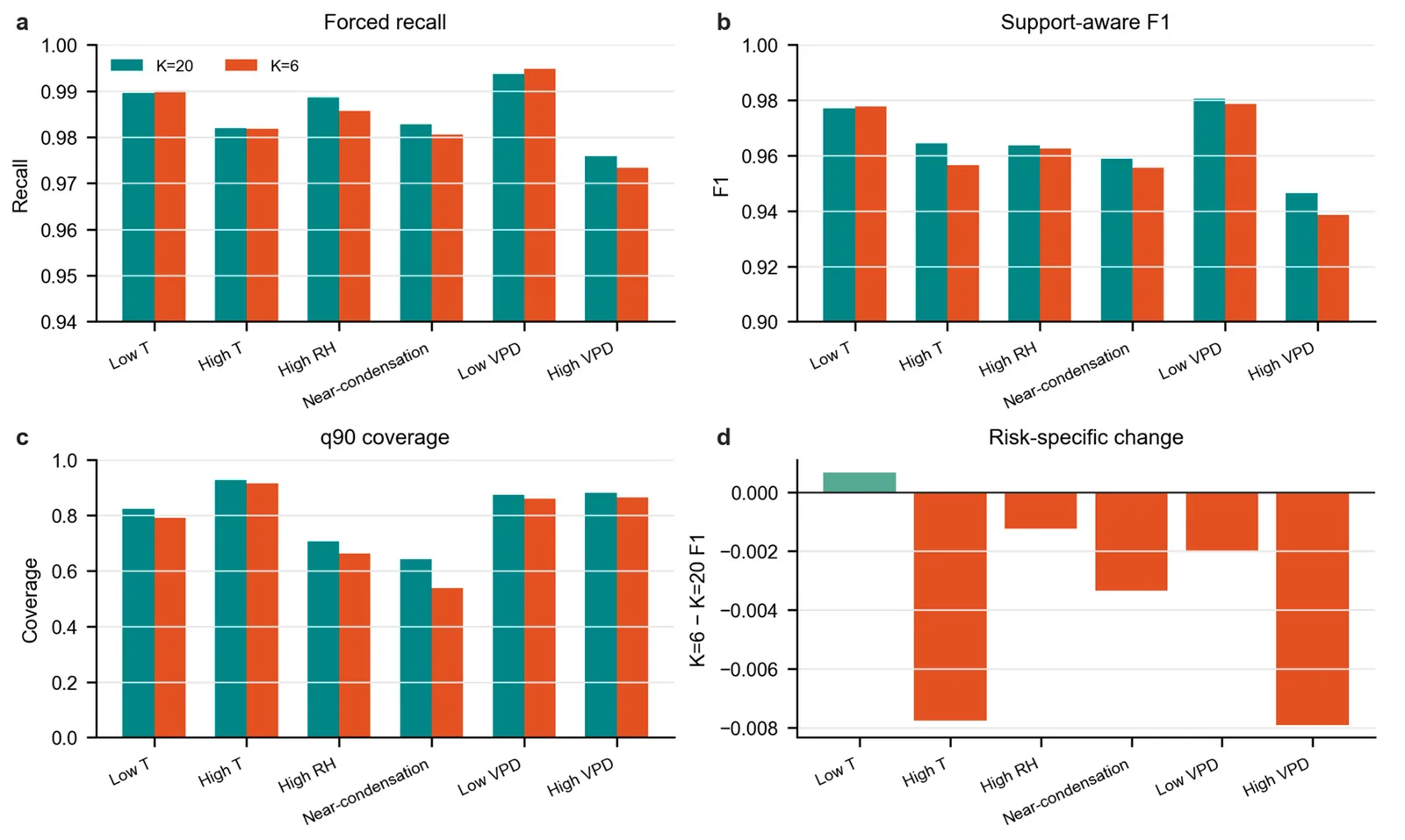
Final-test risk-specific fidelity and selective coverage. (**a**) Forced recall. (**b**) Support-aware F1. (**c**) Development-calibrated 90th-percentile (q90) retained-decision coverage. Teal and orange bars in (**a**–**c**) indicate K = 20 and K = 6, respectively. (**d**) Risk-specific support-aware F1 differences (K = 6 minus K = 20); sage and orange bars indicate positive and negative differences, respectively. T, temperature; RH, relative humidity; VPD, vapour pressure deficit.

The macro metrics did not conceal the collapse of any individual risk category. At K = 6, forced recall ranged from 0.973 for high VPD to 0.995 for low VPD, and support-aware F1 ranged from 0.939 for high VPD to 0.979 for low VPD. The remaining risks also retained high forced-decision performance: low-temperature recall and support-aware F1 were 0.990 and 0.978, respectively; high-temperature values were 0.982 and 0.957; high-humidity values were 0.986 and 0.963; and near-condensation-potential values were 0.981 and 0.956. The risk-specific fidelity and selective coverage are shown in Figure 4 and Table 3. Supplementary Table S2 shows that event support varied by stage and risk, including lower fruit-set prevalence for high temperature (8.0%) and high VPD (8.2%); therefore, risk-specific results should be interpreted alongside their observed event support. Thus, the sparse network preserved all six predefined risk decisions in the forced-decision evaluation.

Next, we applied the development-calibrated q90 risk-margin rule as a secondary selective-inference analysis. The mean q90 coverage at K = 6 was 0.773, compared with 0.809 at K = 20, a decrease of 0.036. Coverage was risk-dependent: it was 0.663 for high humidity and 0.538 for near-condensation potential, whereas it was 0.917 for high temperature and 0.865 for high VPD. This separation between forced-decision fidelity and retained-decision coverage indicates that a high support-aware F1 does not imply equally high confidence for every risk state. Therefore, the selective-inference result supports an operating-limit claim, rather than replacing the primary sparse-network result.

### 3.5. The Sparse-Network Result Was Robust to the Predefined Agricultural Thresholds

**Figure 5:**
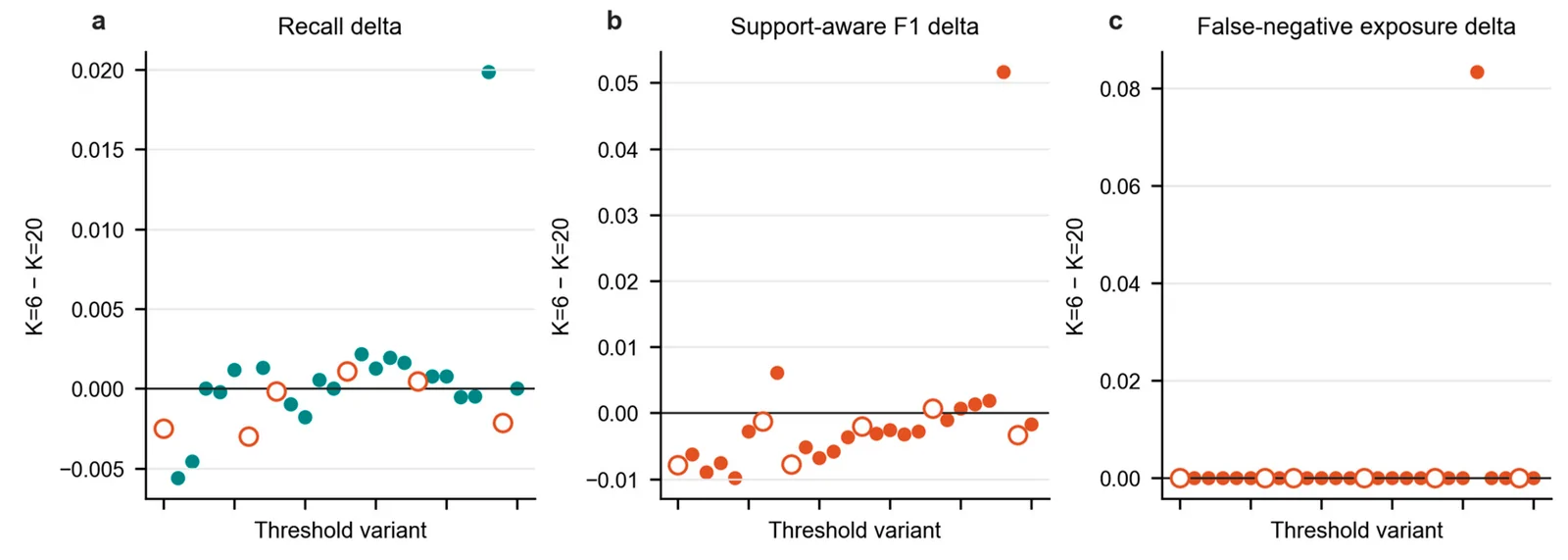
Final-test robustness to agricultural-threshold variants. K = 6 minus K = 20 differences in (**a**) recall (teal), (**b**) support-aware F1 (orange), and (**c**) false-negative exposure (orange) across 26 predefined variants. Open circles identify the primary threshold set, and filled circles identify one-factor sensitivity variants.

We tested 26 predefined one-factor threshold variants covering the stage-specific temperature, relative humidity, VPD, and dew-point-depression boundaries. The estimator and network-selection rule were fixed under the primary validation definition and were not re-selected for individual threshold variants. Across these variants, the worst K = 6 minus K = 20 recall difference was −0.006, the worst support-aware F1 difference was −0.010, and the maximum increase in false-negative exposure was +0.083 h per day. The final-test sensitivity pattern is shown in Figure 5, and the complete values are reported in the threshold-sensitivity source tables. All threshold-sensitivity safeguards passed. The final-test result therefore did not depend on a single agricultural threshold combination within the prespecified sensitivity family.

### 3.6. Relative Performance Was Stable Across Calendar Blocks, Although Absolute B20 Fidelity Was Lower

**Figure 6:**
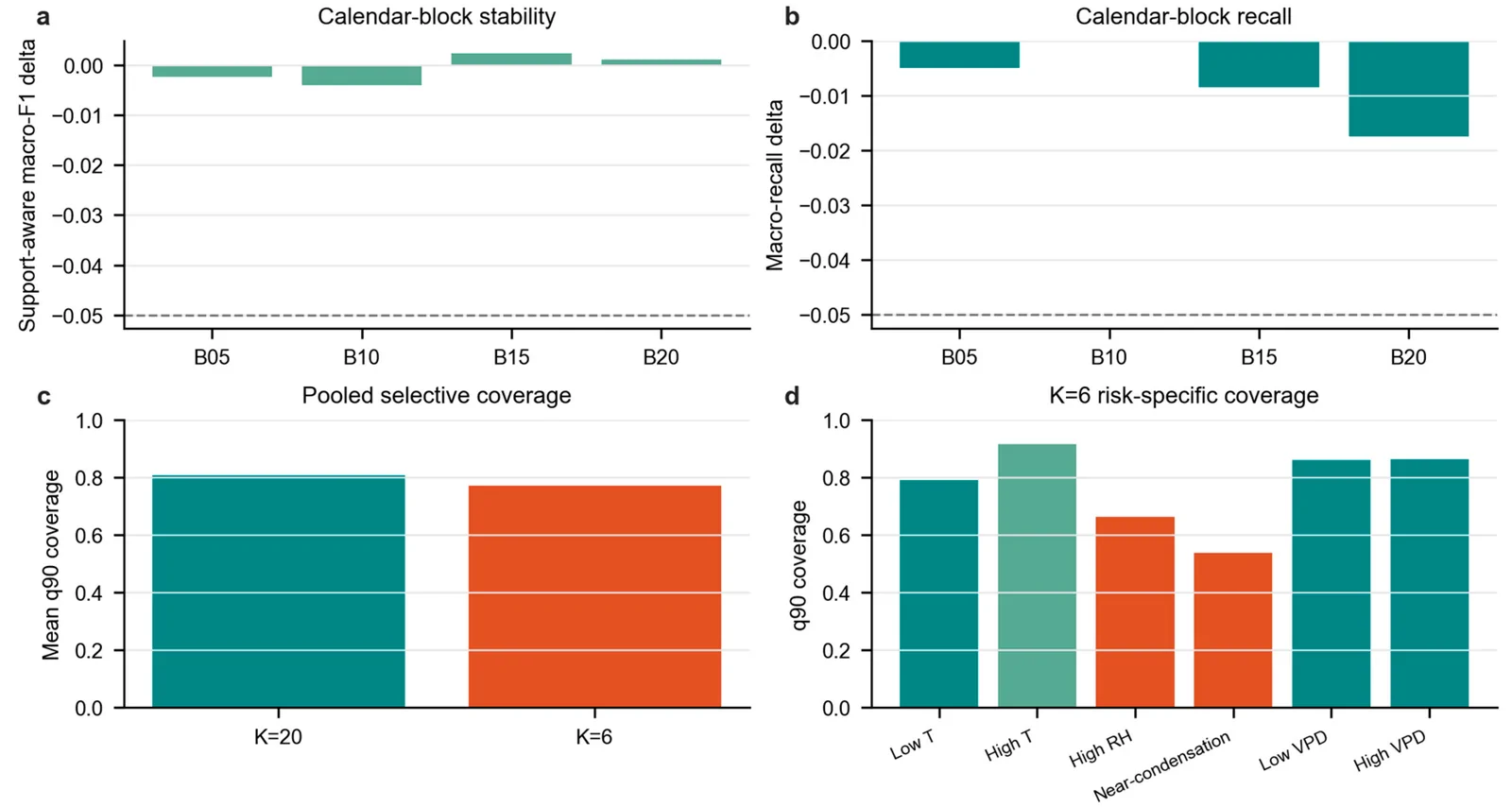
Final-test block stability and selective operating limits. Calendar-block differences (K = 6 minus K = 20) in (**a**) support-aware macro-F1 (sage) and (**b**) macro-recall (teal); grey dashed lines mark −0.05. (**c**) Pooled development-calibrated 90th-percentile (q90) retained-decision coverage for K = 20 (teal) and K = 6 (orange). (**d**) K = 6 risk-specific q90 coverage; colours distinguish high temperature (sage), high relative humidity and near-condensation potential (orange), and the remaining risks (teal).

The four-block analysis showed that the pooled result was not driven exclusively by one calendar block (Figure 6). The K = 6 minus K = 20 support-aware macro-F1 differences were −0.002, −0.004, +0.002 and +0.001 in B05, B10, B15 and B20, respectively. All four blocks met the prespecified −0.05 relative F1-stability criterion, and none fell below the −0.10 floor. B20 nevertheless had substantially lower absolute support-aware macro-F1 than the other blocks: 0.643 at K = 20 and 0.644 at K = 6. High-humidity and near-condensation positive events were sparse in this block, so the low absolute value is interpreted as a shared event-support limitation rather than demonstrated K = 6-specific degradation. We therefore retain the pooled conclusion that K = 6 was confirmed while treating the B20 result as an important limitation on uniform absolute performance and generalisation. The complete block-level values are reported in Supplementary Table S6.

#### Results-to-Claim Boundary

Together, these results support the bounded conclusion that six longitudinally and vertically balanced functioning non-target indoor T/RH inputs can preserve temperature, humidity, VPD, dew-point-depression, and six-risk inference close to a 20-input network in the studied Chinese solar greenhouse and tested fruit-set and fruiting periods. Support-aware scoring retained the K = 6 decision-fidelity conclusion while exposing a shared low-absolute-performance period when risk-event support was sparse. The evidence does not establish that arbitrary six-sensor layouts are sufficient, that the result generalises to other greenhouses or untested growth stages, or that sparse sensing improves yield, disease outcomes, energy use, or closed-loop control. The near-condensation endpoint is a threshold-defined risk state and should not be described as a direct observation of condensation.

## 4. Discussion

### 4.1. The Central Advance Is Risk-Preserving Sparsification with an Explicit Layout Constraint

The central result is that a greenhouse T/RH network can retain risk-relevant information after substantial sensor loss when the remaining inputs satisfy a defined longitudinal and vertical coverage constraint. That constraint is physically plausible because recent field and modelling studies show that greenhouse temperature and humidity fields vary with solar forcing, crop effects, ventilation, structure, and measurement height [7,8,9,10,11]. Existing greenhouse sensor-placement studies have shown that reduced subsets can reproduce a network-level temperature or humidity reference [16,17], whereas greenhouse fault management and imputation work has focused on detecting, correcting, or reconstructing faulty and missing measurements [12,21,22]. Under whole-sensor holdout and a frozen transparent estimation policy, six longitudinally and vertically balanced non-target inputs preserved continuous reconstruction and six stage-aware risk decisions close to the 20-input reference in the sealed final test. This reframes sparse sensing as a decision-preservation problem: the operational question is not only how accurately a missing value can be reconstructed, but whether the reduced network still supports reliable judgements about low temperature, excessive humidity, VPD stress, and near-condensation potential.

The additional validation comparison clarifies the role of layout geometry. Longitudinally and vertically balanced K = 6 layouts consistently outperformed deliberately concentrated K = 6 layouts across all four tested endpoints and four prespecified validation blocks. This result supports longitudinal and vertical coverage as a robustness constraint: preserving longitudinal and vertical representation reduces the degradation that occurs when the available inputs are concentrated in only a small part of the greenhouse. The effect was clearest for temperature and RH reconstruction, but was also detectable in macro-recall and macro-F1. Because the concentrated layouts were intentionally geometry-poor, this analysis demonstrates protection against a meaningful adverse layout condition rather than estimating the frequency of such layouts in all real failure scenarios.

A comparison with unconstrained random layouts provides an equally important boundary. Longitudinally and vertically balanced layouts improved continuous reconstruction and showed a small macro-recall advantage, whereas the macro-F1 interval crossed zero. Therefore, the present evidence does not justify the claim that longitudinal and vertical coverage universally improves every risk metric relative to arbitrary random layouts. Threshold-defined risk decisions can remain stable despite modest numerical reconstruction differences, particularly when estimates remain on the same side of the decision boundary. Consequently, longitudinal and vertical coverage should be presented as a design condition that reduces vulnerability to concentrated loss, rather than as a universally dominant sensor-selection algorithm.

Table 4 shows why the published error values should not be treated as a common performance ranking. Studies that reduced 56 locations to 8–10 sensors or 22 locations to four sensors generally reconstructed a greenhouse-wide aggregate or selected optimal locations and reported lower continuous errors than the present K = 6 local-target reconstruction [16,17,18,19,23]. The 1D-CNN fault-imputation study reported indoor temperature and RH RMSE values of 0.86 °C and 3.47%, which are numerically close to the present K = 6 MAE values of 0.806 °C and 3.526 percentage points [20]; however, RMSE and MAE differ, and that study predicted one environmental index from other indices rather than withholding each spatially paired node. The present method is therefore not claimed to be a universally superior continuous predictor. Its distinct contribution is the all-target complete-pair holdout and the evaluation of whether six stage-aware risk decisions, false-negative exposure and an abstention boundary remain stable as functioning spatial inputs are reduced. None of the compared studies reported this combined decision-fidelity endpoint.

### 4.2. Risk Fidelity Complements, but Does Not Replace, Continuous Reconstruction Error

The final-test K = 6 versus K = 20 comparison showed only small increases in temperature and RH error, accompanied by small changes in macro-recall and support-aware macro-F1, and no increase in median false-negative exposure. Because every K-level comparison used the same held-out target measurements, the K = 6 minus K = 20 differences quantify paired algorithmic degradation against a common measurement reference; they should not be interpreted as resolving absolute environmental differences below the manufacturer-specified instrument accuracy. The support-aware calculation also changed the interpretation of B20: rather than indicating K = 6-specific F1 degradation, it identified low absolute macro-F1 shared by K = 20 and K = 6 under sparse high-humidity and near-condensation event support. The layout comparison explains why continuous and decision endpoints should be interpreted together. Continuous error is sensitive to the geometry of the retained inputs, whereas threshold-based risk labels may be less sensitive when reconstruction differences do not cross the relevant agricultural boundary. A network evaluation based only on MAE could therefore overstate the practical consequence of minor error changes, whereas risk metrics alone could conceal a loss of numerical fidelity or limited event support. Reporting both types of endpoint is necessary to define a usable sparse-sensing operating condition. The primary final-test summaries retain the frozen aggregation and acceptance gates. The added paired-date analysis uses dates, rather than 5 min bins, targets or subset replicates, as the inferential units and reports stratified-date and whole-block bootstrap intervals, exact sign tests and leave-one-block-out sensitivity. The mostly zero-crossing intervals and non-significant sign tests do not prove equivalence or non-inferiority; they show that no stable directional shift was detected at the date level while the prespecified preservation gates remained satisfied. With only four calendar blocks, whole-block resampling is interpreted as a sensitivity check rather than evidence of independent experimental replication or a basis for a superiority claim.

The 26-threshold sensitivity analysis further limits dependence on one set of crop-environment boundaries. The worst K = 6 minus K = 20 support-aware F1 difference was −0.010, and all variants remained within the prespecified preservation limits across the tested temperature, RH, VPD, and dew-point-depression boundaries. This should be interpreted as robustness within a specified agricultural threshold family, not as validation of universal thresholds across cultivars, seasons, or greenhouse management strategies.

### 4.3. Selective Inference Identifies a Complementary Operational Boundary

Forced-decision performance remained high at K = 6, but the q90 selective-inference analysis retained fewer decisions for high humidity and near-condensation potential. Selective classification formalises the trade-off between accepted-decision coverage and error [33,34,35]. This distinction is operationally useful because a system that always emits a risk label can appear reliable while concealing uncertainty near humidity-related decision boundaries. The selective layer is therefore best understood as an uncertainty-boundary diagnostic: a rejected decision could motivate additional sensing, a conservative response, or delayed action; however, those downstream actions were not tested in this study.

### 4.4. Scope and Limitations

Several limitations bound the contribution. First, this was intentionally a single-greenhouse case study of information preservation under progressive loss of inputs from a fixed dense sensor network. Holding the greenhouse structure, sensor geometry, target measurements, and operating context constant supported internally paired comparisons among K levels, but it does not establish transferability to other structures or climates. Published heterogeneity patterns vary with solar forcing, crop characteristics, ventilation, greenhouse form, and sensor position [7,8,9,10,11], reinforcing the need for external validation rather than supporting direct transfer of the present six-input result. The four non-overlapping test blocks provided temporally separated checks within the observed period, not independent experimental replicates. Because they comprised one fruit-set block and three fruiting blocks, they also do not establish repeatability across all crop stages or seasons. Second, complete target-sensor holdout evaluates whole-sensor information loss, not bias drift, intermittent communication, or correlated physical failures. Whole-sensor holdout was activated by design and did not detect failures from measurements. A deployable workflow would require a separately validated diagnostic layer before reconstruction begins. That layer should identify complete unavailability and distinguish it from communication interruption, bias drift, and transient anomalies. Such diagnostic functions were outside the scope of the present study and remain a requirement for future deployment. Although all transmitters shared the same model and manufacturer and were obtained in one procurement batch, no independent pre-installation co-location calibration was performed. The analysis consequently treats held-out target sensors as measurement references and does not propagate manufacturer accuracy, unit-to-unit variation, or calibration uncertainty into the reconstruction and risk metrics. Third, the new layout comparison was exploratory and validation-only. It used prespecified calendar blocks and paired block resampling but was introduced after the original final-test protocol. It should not be described as part of the prespecified confirmation. Fourth, spatially concentrated layouts are a deliberately adverse geometry class rather than an estimate of the distribution of all real sensor-failure patterns. No transverse-position coverage rule was imposed; accordingly, the selected subsets should not be interpreted as balanced across all three spatial dimensions. Fifth, B20 had low absolute support-aware macro-F1 at both K = 20 and K = 6 because some risk-positive events were sparse. This limits claims of uniformly high absolute fidelity over time even though relative K = 6 preservation passed. Finally, the risk states were threshold-defined indicators, not direct measurements of condensation, disease, yield, energy use, or closed-loop control performance.

The present K = 6 result describes a degraded-operation information state, not a recommended six-sensor installation. It asks whether monitoring can continue after extensive loss from the existing 22-pair reference network. The retained six inputs must still satisfy the tested longitudinal and vertical coverage rule. They do not establish that a new greenhouse should be equipped with only six sensors. We did not collect installation, maintenance, downtime, labour, or lifecycle-cost data. Therefore, the study cannot quantify cost savings or balance financial savings against spatial information loss. In practice, the tested state could support temporary continuity while failed sensors are inspected or replaced, but this operational response was not evaluated. Independent multi-greenhouse validation, realistic fault injection, and economic analysis are needed before any deployment or cost recommendation.

## 5. Conclusions

This study evaluated whether threshold-defined agronomic risk inference could be preserved after extensive loss of paired indoor T/RH sensors. Four specific conclusions follow.

(1) Under complete target-sensor holdout and a frozen estimation policy, reducing the functioning non-target inputs from K = 20 to six longitudinally and vertically balanced pairs increased temperature MAE by 0.014 °C and RH MAE by 0.027 percentage points. Macro-recall changed by −0.001, support-aware macro-F1 changed by −0.004, and median false-negative exposure remained 0.083 h/day. The K = 6 condition therefore passed all prespecified absolute-usability and relative-preservation gates in the sealed final test.
(2) The preservation result was not tied to one analysis scale or one agricultural threshold combination. Across 28 paired test dates, the median K = 6 minus K = 20 support-aware macro-F1 difference was −0.003 (stratified-date 95% interval, −0.005 to 0.000; exact sign-test *p* = 0.122). All 26 predefined threshold variants also remained within the preservation limits, with a worst risk-specific support-aware F1 difference of −0.010. These analyses support bounded robustness, but they are not evidence of statistical equivalence, non-inferiority, or universal agricultural thresholds.
(3) Sensor geometry and event support constrained performance. Spatially concentrated K = 6 layouts had higher temperature and RH errors and lower macro-F1 than the longitudinally and vertically balanced layouts, although the balanced layouts did not show a consistent macro-F1 advantage over unconstrained random layouts. All four relative block-stability checks passed, but B20 had low absolute macro-F1 at both K = 20 and K = 6 because relevant positive events were sparse. Longitudinal and vertical coverage should therefore be treated as protection against concentrated input loss, not as a universally optimal sensor-selection rule.
(4) The confirmed K = 6 condition represents a temporary degraded-operation information state after substantial sensor loss, not a recommended six-sensor installation or evidence of cost savings. The evidence is limited to one greenhouse, complete whole-sensor holdout, the observed crop stages and threshold-defined risk indicators; it does not validate bias-drift detection, intermittent failures, biological outcomes, closed-loop control or economic performance. Independent validation across greenhouse types, crop stages, realistic fault modes and economic conditions is required before deployment or cost recommendations.

## Figures and Tables

**Table 1:** Frozen study design and final test evaluation structure.

Component	Frozen Specification
Study setting	One Chinese solar greenhouse
Indoor sensing modality	Paired indoor temperature and relative humidity (T/RH)
Retained target pairs	22
Holdout type	Complete whole-sensor target holdout
Functioning-input counts	K = 20, 16, 12, 8 and 6
Target-specific subsets	20 deterministic subsets for every target and K
Longitudinal and vertical coverage constraint	All four sections and three nominal heights represented; no section or height >50% of inputs
Frozen estimator	Same-height median at K = 20, 16 and 12; whole-network median at K = 8 and 6
Direct reconstruction variables	Temperature and RH
Derived variables	Vapour pressure deficit (VPD) and dew-point depression (DPD)
Evaluated risks	Low temperature, high temperature, high RH, near-condensation potential, low VPD and high VPD
Final-test dates	28 dates in four non-overlapping seven-day blocks
Final-test stages	One fruit-set block and three fruiting blocks
Threshold variants	26 predefined one-factor variants
Selective inference	Development-calibrated q90 abstention rule
Outdoor variables in final test	Not used
Primary outcome	CONFIRMED_K6

Abbreviations: T/RH, temperature and relative humidity; K, functioning non-target input-pair count; VPD, vapour pressure deficit; DPD, dew-point depression; q90, development-calibrated 90th-percentile abstention rule. CONFIRMED_K6 indicates that the six-input network met the prespecified acceptance gates.

**Table 2:** Final-test comparison between the 20-input and six-input networks. Differences were calculated from unrounded values; therefore, displayed values may not subtract exactly.

Metric	K = 20	K = 6	K = 6–K = 20	Prespecified Gate	Gate
Temperature MAE (°C)	0.792	0.806	+0.014	≤+0.20 °C relative	Pass
RH MAE (percentage points)	3.499	3.526	+0.027	≤+1.00 pp relative	Pass
VPD MAE (kPa)	0.156	0.175	+0.019	Secondary diagnostic	—
DPD MAE (°C)	0.953	0.983	+0.030	Secondary diagnostic	—
Forced macro-recall	0.985	0.984	−0.001	≥−0.03 relative	Pass
Support-aware macro-F1	0.965	0.962	−0.004	≥−0.03 relative	Pass
Median false-negative exposure (h/day)	0.083	0.083	+0.000	≤+0.25 h/day relative	Pass
Mean q90 coverage	0.809	0.773	−0.036	≥−0.10 secondary	Pass

Values are the final test medians or means, as defined in the Materials and Methods. MAE, mean absolute error; RH, relative humidity; VPD, vapour pressure deficit; DPD, dew-point depression; pp, percentage points. K, functioning non-target input-pair count; q90, development-calibrated 90th-percentile abstention rule.

**Table 3:** Risk-specific final test fidelity and selective coverage at K = 6.

Risk	Forced Recall	Support-Aware F1	False-Negative Exposure (h/day)	q90 Coverage	Risk Safeguard
Low temperature	0.990	0.978	0.083	0.792	Pass
High temperature	0.982	0.957	0.083	0.917	Pass
High humidity	0.986	0.963	0.000	0.663	Pass
Near-condensation potential	0.981	0.956	0.000	0.538	Pass
Low VPD	0.995	0.979	0.083	0.861	Pass
High VPD	0.973	0.939	0.083	0.865	Pass

Risk safeguards required recall ≥0.80, support-aware F1 ≥ 0.75, and false-negative exposure ≤1.0 h/day. Near-condensation potential is a threshold-defined state, not direct observation of condensation. VPD, vapour pressure deficit; K, functioning non-target input-pair count; q90, development-calibrated 90th-percentile abstention rule.

**Table 4:** Structured comparison with greenhouse sensor-reduction, placement and fault-reconstruction studies.

Study	Sensing Design	Method and Target	Reported Result	Comparison with Present Study	Why Not Directly Rankable
van Mourik et al. (2019) [32]	Single sensors assessed against grid averages in two greenhouses; expected 1-to-25-sensor effect discussed	EKF, UKF and moving-average filtering; compartment-average climate	Unfiltered RMSE 0.43/0.80 °C and 0.48/0.64 g m^−3^; filters did not improve accuracy overall	Lower reported T error than K = 6 MAE	Aggregate target; humidity unit differs; no complete local holdout or risk endpoint
Uyeh et al. (2022) [18]	56 paired locations; monthly selected counts varied (T 3–13; RH 2–7)	Gradient boosting plus grid/random search; one-step forecasting and Pareto selection	<32% of 56 sensors; count and RMSE optimum varied by month	Lower reported RMSE	Different forecast target and monthly selection; no all-target holdout or risk endpoint
Ajani et al. (2023) [16]	56 paired sensors reduced to 8	Genetic-programming symbolic regression; weighted network reference	Mean RMSE 0.082 °C and 0.253 RH units; mean r ≈ 0.999	Lower reported continuous error	Aggregate reference and random split; no complete local holdout or risk endpoint
Ajani et al. (2023) [17]	56-location data; 10 fixed inputs predicted 10 ranked target locations	DNN cross-month T/RH prediction	Average RMSE reduction 68.67% (T) and 46.21% (RH) versus fixed readings without DNN	Compact sensing supported; absolute ranking unavailable	Percentage reduction and selected-location forecast differ from local-target MAE
Song et al. (2024) [19]	22 T/RH sensors reduced to 4 in a Chinese solar greenhouse	Weighted HSIC ranking with RMSE and information-gain thresholds	Maximum RMSE 0.6 °C and 2.30% RH	Lower reported continuous error and fewer sensors	Original monitoring solution is the reference; no complete local holdout, threshold variants or risk endpoint
Shekarian et al. (2024) [20]	Six environmental indices; one faulty index predicted from five remaining indices	1D CNN, decision tree and linear regression; variable-level fault imputation	Indoor T/RH RMSE 0.86 °C and 3.47% RH	Numerically similar to K = 6 T/RH MAE	RMSE versus MAE and variable-level versus spatial-node targets differ; no risk endpoint
Akintan et al. (2025) [23]	56 paired sensors; top 10 compared with full network	Offline Thompson-sampling ranking in a digital twin	Example full/reduced means differed by 0.4 °C and 0.5% RH	Lower aggregate mean differences	Aggregate seasonal ranking, not fixed all-target holdout or risk preservation
Present study	22 eligible paired sensors; every target held out; K = 20 to K = 6 non-target inputs	Frozen median policy plus longitudinal/vertical coverage; local T/RH and six risks	K = 6 MAE 0.806 °C and 3.526 pp; macro-F1 0.962; false-negative exposure 0.083 h/day	Continuous error higher than aggregate-target studies and close to CNN fault imputation	Adds all-target complete-pair holdout, threshold sensitivity, risk fidelity and abstention; one-greenhouse scope

Abbreviations: EKF, extended Kalman filter; UKF, unscented Kalman filter; DNN, deep neural network; HSIC, Hilbert–Schmidt independence criterion; CNN, convolutional neural network; 1D, one-dimensional; RMSE, root mean squared error; MAE, mean absolute error; T, temperature; RH, relative humidity; T/RH, temperature and relative humidity; pp, percentage points; K, functioning non-target input-pair count; r, correlation coefficient.

## Data Availability

Aggregated final-test summaries, source data underlying Figure 1, Figure 2, Figure 3, Figure 4, Figure 5 and Figure 6, the target-specific subset catalogue, analysis scripts and the execution manifest are available from the corresponding authors upon reasonable request. Raw project observations, detailed site metadata and access-dependent code are not publicly released because they form part of continuing site-specific monitoring and related analyses; they can be made available to editors and reviewers for manuscript verification under controlled access. Requests should be directed to the corresponding authors.

## Supplementary Materials

The following supporting information can be downloaded at: https://www.mdpi.com/article/10.3390/s26206391/s1, Table S1, Traceability of agricultural decision thresholds; Table S2a, Final-test risk-event support, fruit set stage; Table S2b, Final-test risk-event support, fruiting stage; Table S3, Basis and outcomes of the acceptance gates; Table S4, Original sensor coordinates, mounting context and fixed-reference status; Table S5, Complete 26-variant threshold sensitivity; Table S6, Block-level decision performance; Table S7, Paired date-level uncertainty and leave-one-block-out sensitivity. References [14,15,26,27,28,29,30] are cited in the Supplementary Materials.
