# Supplementary material for: Longitudinally and Vertically Balanced Sparse Indoor Temperature–Humidity Sensing Preserves Threshold-Defined Risk Inference in a Chinese Solar Greenhouse
Source: Sensors (Basel). 2026 Oct 9;26(20):6391. doi: 10.3390/s26206391 (PMC13708978; doi:10.3390/s26206391)
Supplement: Supplementary file 1 [file sensors-26-06391-s001.zip › sensors-4503602-supplementary.pdf]

Supplementary Materials

Longitudinally and vertically balanced sparse indoor temperature–humidity sensing preserves threshold-defined risk inference in a Chinese solar greenhouse

Supplementary Table S1 documents the provenance and intended interpretation of the threshold-defined risk states, and Supplementary Table S2 documents final-test risk-event support. All rules were prespecified operational decision rules for evaluating risk fidelity; they were not fitted to the sealed final test and do not claim a universal physiological optimum. Supplementary Table S3 gives the basis, frozen requirement and corrected support-aware outcome for each acceptance-gate family. Supplementary Table S4 reports the original 24-pair installation, mounting context, health-cycle coverage, and the pre-analysis exclusion of pairs 20A and 20C. Supplementary Table S5 reports all 26 prespecified threshold variants and their K=20 and K=6 decision metrics. Supplementary Table S6 reports performance for each of the four final-test blocks. Supplementary Table S7 reports paired date-level uncertainty and leave-one-block-out sensitivity for the five primary K=6-minus-K=20 endpoints.

Supplementary Table S1. Traceability of agricultural decision thresholds.

Risk rule	Traceability, evidence and manuscript boundary
Low/high temperature<br>17–26 °C (fruit set)	A prespecified project decision rule informed by stage-specific tomato production guidance [26,27]. The cited books provide production context but are not quoted as the unique direct source of the exact numerical band.
Low/high temperature<br>18–27 °C (fruiting)	The corresponding prespecified operational rule for the monitored fruiting period, informed by stage-specific tomato production guidance [26,27]. Threshold variants quantify whether the sparse-network conclusion depends on this boundary. The cited books provide contextual production support rather than direct validation of sparse-sensor performance.
Low/high VPD<br>0.8–1.2 kPa (fruit set)	Shamshiri et al. [14] reported flowering-to-mature-fruiting tomato VPD optimal bounds that vary with light condition: 0.596–1.425 kPa in sun, 0.528–1.193 kPa in cloud and 0.413–0.935 kPa at night. The band is therefore a compact daytime operational screen, not a universal all-day optimum.
Low/high VPD<br>0.8–1.3 kPa (fruiting)	The 1.3 kPa upper boundary is within the reported sun-condition optimum but above the cloud and night optima [14]. It is retained as a pragmatic high-VPD screening boundary and tested in sensitivity analysis. Zhang et al. [15] independently reported the agronomic relevance of reducing excessive VPD in greenhouse tomato.
High RH<br>RH >85%	A high-humidity risk screen, not a diagnosis of disease. Li et al. [29] reported successful tomato grey-mould infection when RH >85% persisted for more than 8 h under their study conditions. Schwarz et al. [28] identified prolonged RH >85% with high temperature as critical for fruit set and noted enhanced fungal foliar-disease risk.
Near-condensation potential<br>DPD ≤2 °C	A conservative near-condensation-potential screen, not direct observation of condensation or leaf wetness. Wang et al. [30] used a 2 °C dew-point-depression threshold as an empirical dew-presence rule in greenhouse leaf-wetness modelling evaluated in Chinese and Spanish greenhouses.

Note: 'Near-condensation potential' is a threshold-defined state and does not represent direct observation of condensation.

Supplementary Table S2a. Final-test risk-event support, fruit set stage.

Risk	Target-pair days	True-positive 5 min bins	Valid 5 min bins	True-risk exposure (h)	Prevalence (%)
High VPD	154	3,601	43,731	300.1	8.2
High humidity	154	33,609	43,731	2800.8	76.9
High temperature	154	3,502	43,731	291.8	8.0
Low VPD	154	38,440	43,731	3203.3	87.9
Low temperature	154	33,342	43,731	2778.5	76.2
Near-condensation potential	154	32,110	43,731	2675.8	73.4

Supplementary Table S2b. Final-test risk-event support, fruiting stage.

Risk	Target-pair days	True-positive 5 min bins	Valid 5 min bins	True-risk exposure (h)	Prevalence (%)
High VPD	462	46,152	129,517	3846.0	35.6
High humidity	462	41,867	129,517	3488.9	32.3
High temperature	462	39,466	129,517	3288.8	30.5
Low VPD	462	66,496	129,517	5541.3	51.3
Low temperature	462	46,893	129,517	3907.8	36.2
Near-condensation potential	462	34,279	129,517	2856.6	26.5

Support was calculated from one K=20 primary-threshold record per target pair, date and risk after duplicate subset evaluations were removed. Five-minute bins are the observed truth support, not independent replicate counts.

Supplementary Table S3. Basis, frozen requirement and support-aware final-test outcome for the acceptance gates.

Gate family	Frozen requirement and basis	Support-aware final-test outcome
K = 6 absolute continuous error	Temperature MAE ≤ 1.0 °C; RH MAE ≤ 5.0 percentage points. Study-specific engineering tolerances informed by the VMS-3002 nominal reference-condition accuracy (±0.5 °C at 25 °C; ±3% RH at 60% RH and 25 °C) and contextual greenhouse reconstruction scales; not universal agronomic or calibration limits.	Temperature MAE = 0.806 °C; RH MAE = 3.526 percentage points. Both gates passed.
K = 6 absolute decision performance	Macro-recall ≥ 0.85; support-aware macro-F1 ≥ 0.80; median false-negative exposure ≤ 0.5 h day−1. Author-defined anti-masking guardrails; 0.5 h equals six 5 min bins and is not a crop-damage boundary.	Macro-recall = 0.984; support-aware macro-F1 = 0.962; median false-negative exposure = 0.083 h day−1. All gates passed.
K = 6 versus K = 20 continuous preservation	Temperature MAE difference ≤ +0.20 °C; RH MAE difference ≤ +1.00 percentage point. Author-defined margins representing the study's frozen meaning of close-to-reference preservation.	Temperature difference = +0.014 °C; RH difference = +0.027 percentage points. Both gates passed.
K = 6 versus K = 20 decision preservation	Macro-recall difference ≥ −0.03; support-aware macro-F1 difference ≥ −0.03; false-negative-exposure increase ≤ +0.25 h day−1. Author-defined limits intended to prevent a small average change from concealing decision loss.	Recall difference = −0.001; support-aware macro-F1 difference = −0.004; exposure difference = +0.000 h day−1. All gates passed.

Gate family	Frozen requirement and basis	Support-aware final-test outcome
Risk-specific safeguards	For every risk: recall ≥ 0.80, support-aware F1 ≥ 0.75 and false-negative exposure ≤ 1.0 h day−1. Author-defined safeguards against a macro-average masking failure of one risk.	All six risks passed. Minimum recall = 0.973 and minimum support-aware F1 = 0.939 (high VPD); maximum median false-negative exposure = 0.083 h day−1.
Threshold robustness	Across 26 prespecified agricultural-threshold variants: worst recall and F1 differences ≥ −0.03; maximum false-negative-exposure increase ≤ +0.25 h day−1. This tests dependence on operational label definitions, not whether every threshold is agronomically optimal.	All threshold-robustness conditions passed; the worst risk-specific support-aware F1 difference was −0.010.
Block stability	For recall and support-aware F1, at least three of four blocks had K = 6 minus K = 20 ≥ −0.05, and no block was below −0.10. Author-defined temporal anti-collapse rule.	All four blocks passed. Support-aware macro-F1 differences were −0.002 (B05), −0.004 (B10), +0.002 (B15) and +0.001 (B20).
Selective inference	K = 6 mean q90 coverage ≥ 0.70 and K = 6 minus K = 20 coverage ≥ −0.10. Secondary author-defined abstention criteria rather than universal deployment requirements.	SELECTIVE_PASS. The support-aware forced-decision F1 correction did not affect q90 calculations.

Note: The acceptance values are prespecified study criteria, not universal agricultural safety thresholds. Manufacturer accuracy applies only at the stated reference conditions and does not replace co-location calibration. Support-aware F1 = 2TP/(2TP + FP + FN); complete misses receive F1 = 0 whenever the denominator is positive.

Supplementary Table S4. Original T/RH sensor installation, mounting context, and fixed-reference status.

Pair	Position	x (m)	y (m)	z (m)	Relation to canopy top	Valid coverage (%)	Fixed-reference status
10A	A	10	5	0.7	0.6-0.8 m below canopy	94.4	Retained in 22-pair catalogue
10B	B	10	3	1.2	0.1-0.3 m below canopy	96.2	Retained in 22-pair catalogue
10C	C	10	3	0.7	0.6-0.8 m below canopy	96.5	Retained in 22-pair catalogue
10D	D	10	1	1.7	0.2-0.4 m above canopy	96.5	Retained in 22-pair catalogue
10E	E	10	1	1.2	0.1-0.3 m below canopy	96.5	Retained in 22-pair catalogue
10F	F	10	1	0.7	0.6-0.8 m below canopy	96.5	Retained in 22-pair catalogue
20A	A	20	5	0.7	0.6-0.8 m below canopy	0.0	Excluded: no valid paired T/RH
20B	B	20	3	1.2	0.1-0.3 m below canopy	96.4	Retained in 22-pair catalogue
20C	C	20	3	0.7	0.6-0.8 m below canopy	22.9	Excluded: 22.9% coverage (<75%)
20D	D	20	1	1.7	0.2-0.4 m above canopy	96.0	Retained in 22-pair catalogue
20E	E	20	1	1.2	0.1-0.3 m below canopy	96.5	Retained in 22-pair catalogue
20F	F	20	1	0.7	0.6-0.8 m below canopy	96.5	Retained in 22-pair catalogue
30A	A	30	5	0.7	0.6-0.8 m below canopy	96.5	Retained in 22-pair catalogue

Pair	Posi-tion	x (m)	y (m)	z (m)	Relation to canopy top	Valid coverage (%)	Fixed-reference status
30B	B	30	3	1.2	0.1-0.3 m below canopy	96.2	Retained in 22-pair catalogue
30C	C	30	3	0.7	0.6-0.8 m below canopy	96.3	Retained in 22-pair catalogue
30D	D	30	1	1.7	0.2-0.4 m above canopy	96.5	Retained in 22-pair catalogue
30E	E	30	1	1.2	0.1-0.3 m below canopy	96.4	Retained in 22-pair catalogue
30F	F	30	1	0.7	0.6-0.8 m below canopy	96.3	Retained in 22-pair catalogue
40A	A	40	5	0.7	0.6-0.8 m below canopy	95.8	Retained in 22-pair catalogue
40B	B	40	3	1.2	0.1-0.3 m below canopy	94.2	Retained in 22-pair catalogue
40C	C	40	3	0.7	0.6-0.8 m below canopy	96.0	Retained in 22-pair catalogue
40D	D	40	1	1.7	0.2-0.4 m above canopy	95.7	Retained in 22-pair catalogue
40E	E	40	1	1.2	0.1-0.3 m below canopy	95.9	Retained in 22-pair catalogue
40F	F	40	1	0.7	0.6-0.8 m below canopy	96.1	Retained in 22-pair catalogue

Notes: All 24 T/RH probes were enclosed in louvred radiation shields. Tomato canopy height was approximately 1.3–1.5 m. The upper ventilation opening was approximately 3.2 m above ground, giving nominal vertical separations of 2.5, 2.0, and 1.5 m from the 0.7, 1.2, and 1.7 m sensor levels, respectively. These distances describe mounting geometry; direct airflow effects were not independently quantified. Coverage was calculated against all expected five-minute bins in the 151-date health-cycle calendar. Coordinates use the project frame with x=0 m at the east end, y=0 m at the north side, and z=0 m at ground level. The probes were VMS-3002-WS-V10 transmitters (VEMSEE, Jinan, China), connected to the DAQ970A acquisition system (Keysight Technologies, Santa Rosa, CA, USA; sourced from Shenzhen, China) and represented on a 5 min grid. All units were purchased in one batch and installed as one network; no independent pre-installation co-location calibration was performed.

Supplementary Table S5. Complete sensitivity of decision performance across 26 prespecified threshold variants. Differences were calculated from unrounded values; therefore, displayed values may not subtract exactly.

Risk	Threshold variant	Primary	Recall K20 / K6 / delta	Support-aware F1 K20 / K6 / delta	Median FN exposure (h) K20 / K6 / delta
High VPD	VPD upper offset +0.0 kPa	Yes	0.976 / 0.973 / -0.002	0.947 / 0.939 / -0.008	0.083 / 0.083 / 0.000
High VPD	VPD upper offset +0.1 kPa	No	0.974 / 0.969 / -0.006	0.949 / 0.943 / -0.006	0.083 / 0.083 / 0.000
High VPD	VPD upper offset +0.2 kPa	No	0.972 / 0.967 / -0.005	0.952 / 0.943 / -0.009	0.083 / 0.083 / 0.000
High VPD	VPD upper offset -0.1 kPa	No	0.977 / 0.977 / 0.000	0.949 / 0.942 / -0.008	0.083 / 0.083 / 0.000
High VPD	VPD upper offset -0.2 kPa	No	0.982 / 0.982 / -0.0002	0.958 / 0.948 / -0.010	0.083 / 0.083 / 0.000
High RH	RH > 80%	No	0.989 / 0.990 / +0.001	0.975 / 0.972 / -0.003	0.000 / 0.000 / 0.000
High RH	RH > 85%	Yes	0.989 / 0.986 / -0.003	0.964 / 0.963 / -0.001	0.000 / 0.000 / 0.000
High RH	RH > 90%	No	0.968 / 0.970 / +0.001	0.913 / 0.919 / +0.006	0.000 / 0.000 / 0.000
High temperature	Temperature upper offset +0 °C	Yes	0.982 / 0.982 / -0.0002	0.964 / 0.957 / -0.008	0.083 / 0.083 / 0.000
High temperature	Temperature upper offset +1 °C	No	0.978 / 0.977 / -0.001	0.960 / 0.955 / -0.005	0.083 / 0.083 / 0.000
High temperature	Temperature upper offset +2 °C	No	0.978 / 0.976 / -0.002	0.962 / 0.955 / -0.007	0.000 / 0.000 / 0.000
High temperature	Temperature upper offset -1 °C	No	0.985 / 0.986 / +0.001	0.971 / 0.965 / -0.006	0.083 / 0.083 / 0.000
High temperature	Temperature upper offset -2 °C	No	0.984 / 0.984 / 0.000	0.971 / 0.968 / -0.004	0.083 / 0.083 / 0.000
Low VPD	VPD lower offset +0.0 kPa	Yes	0.994 / 0.995 / +0.001	0.981 / 0.979 / -0.002	0.083 / 0.083 / 0.000
Low VPD	VPD lower offset +0.1 kPa	No	0.992 / 0.994 / +0.002	0.981 / 0.978 / -0.003	0.083 / 0.083 / 0.000
Low VPD	VPD lower offset +0.2 kPa	No	0.994 / 0.995 / +0.001	0.981 / 0.978 / -0.003	0.083 / 0.083 / 0.000
Low VPD	VPD lower offset -0.1 kPa	No	0.993 / 0.995 / +0.002	0.979 / 0.975 / -0.003	0.083 / 0.083 / 0.000
Low VPD	VPD lower offset -0.2 kPa	No	0.993 / 0.994 / +0.002	0.979 / 0.976 / -0.003	0.083 / 0.083 / 0.000
Low temperature	Temperature lower offset +0 °C	Yes	0.990 / 0.990 / +0.0005	0.977 / 0.978 / +0.001	0.083 / 0.083 / 0.000
Low temperature	Temperature lower offset +1 °C	No	0.990 / 0.991 / +0.001	0.982 / 0.981 / -0.001	0.083 / 0.083 / 0.000
Low temperature	Temperature lower offset +2 °C	No	0.991 / 0.992 / +0.001	0.984 / 0.984 / +0.001	0.083 / 0.083 / 0.000
Low temperature	Temperature lower offset -1 °C	No	0.992 / 0.991 / -0.001	0.971 / 0.973 / +0.001	0.000 / 0.083 / +0.083
Low temperature	Temperature lower offset -2 °C	No	0.987 / 0.986 / -0.0005	0.961 / 0.963 / +0.002	0.000 / 0.000 / 0.000
Near-condensation potential	DPD ≤ 1 °C	No	0.909 / 0.929 / +0.020	0.667 / 0.718 / +0.052	0.000 / 0.000 / 0.000
Near-condensation potential	DPD ≤ 2 °C	Yes	0.983 / 0.981 / -0.002	0.959 / 0.956 / -0.003	0.000 / 0.000 / 0.000

Risk	Threshold variant	Primary	Recall K20 / K6 / delta	Support-aware F1 K20 / K6 / delta	Median FN exposure (h) K20 / K6 / delta
Near-condensation potential	DPD ≤ 3 °C	No	0.989 / 0.989 / 0.000	0.971 / 0.969 / -0.002	0.000 / 0.000 / 0.000

Notes: Values in each metric cell are K=20 / K=6 / K=6-minus-K=20. Temperature and VPD offsets were applied to the stage-specific primary limits in Table S1. RH and DPD variants are absolute cut-offs. FN, false negative. The estimator and subset policy were not reselected for individual variants.

Supplementary Table S6. Block-level final-test decision performance at K=20 and K=6. Differences were calculated from unrounded values; therefore, displayed values may not subtract exactly.

Block	Stage and dates	Recall K20	Recall K6	Delta recall	Support-aware F1 K20	Support-aware F1 K6	Delta F1
B05	Fruit set; 26 Feb-3 Mar 2024	0.981	0.976	-0.005	0.955	0.952	-0.002
B10	Fruiting; 1-7 Apr 2024	0.982	0.982	-0.0002	0.951	0.947	-0.004
B15	Fruiting; 6-12 May 2024	0.961	0.953	-0.009	0.907	0.909	+0.002
B20	Fruiting; 10-16 Jun 2024	0.718	0.700	-0.017	0.643	0.644	+0.001

Notes: Each block comprised seven non-overlapping dates. Delta is K=6 minus K=20. Macro values are unweighted across the six prespecified risks. Lower absolute values in B20 occurred at both network sizes and are interpreted with the event-support information in Table S2.

Supplementary Table S7. Paired date-level uncertainty and leave-one-block-out sensitivity for K=6 versus K=20.

**Metric**	**Paired dates**	**Median delta**	**Stratified-date 95% interval**	**Whole-block 95% interval**	**Exact sign-test p (non-zero n)**	**Leave-one-block-out range**
Temperature MAE (°C)	28	+0.010	−0.040 to +0.030	−0.054 to +0.091	0.851 (28)	−0.035 to +0.028
RH MAE (percentage points)	28	+0.012	−0.065 to +0.151	−0.142 to +0.542	0.851 (28)	−0.022 to +0.066
Macro-recall	28	−0.001	−0.002 to +0.002	−0.001 to +0.001	0.701 (27)	−0.001 to 0.000
Support-aware macro-F1	28	−0.003	−0.005 to 0.000	-0.006 to -9.81×10^{-8}	0.122 (27)	−0.005 to −0.001
Median false-negative exposure (h/day)	28	0.000	0.000 to +0.042	0.000 to +0.021	0.332 (17)	0.000 to 0.000

Notes: Delta is K=6 minus K=20. The date was the inferential unit; five-minute bins, targets, and subset replicates were not treated as independent samples. Percentile intervals used 20,000 deterministic resamples (seed 20,260,905). The stratified-date bootstrap resampled dates within each of the four blocks; the whole-block bootstrap resampled the four complete seven-day blocks. The exact two-sided sign test omitted zero differences; the number of non-zero dates is shown in parentheses. Leave-one-block-out values are medians after excluding each complete block. These are difference-from-zero sensitivity analyses, not equivalence or non-inferiority tests. The value -9.81×10^{-8} is reported rather than rounded to −0.000.
